# Synthesis, Solution, and Structural Characterization of Tetrahydrofuranyl-2,2-Bisphosphonic Acid Disodium Salt

**DOI:** 10.1155/2010/563875

**Published:** 2010-05-11

**Authors:** Elena Maltezou, Marios Stylianou, Sudeshna Roy, Chryssoula Drouza, Anastasios D. Keramidas

**Affiliations:** ^1^Department of Chemistry, University of Cyprus, 1678 Nicosia, Cyprus; ^2^Agricultural Production and Biotechnology and Food Science, Cyprus University of Technology, 3036 Lemesos, Cyprus

## Abstract

Bisphosphonates are biologically relevant therapeutics for bone disorders and cancer. Reaction of *γ*-chlorobutyric acid, phosphorus acid, and phosphorus trichloride without the use of solvent gave the tetrahydrofuranyl-2,2-bisphosphonate sodium salt (Na_2_H_2_L). The Na_2_H_2_L was isolated, characterized in solution by ^1^H, ^13^C, and ^31^P NMR spectroscopy and in solid state by single X-Ray crystallography. The crystal structure showed that the Na_2_H_2_L forms in the crystal infinite two-dimensional sheets stacked one parallel to the other. A comparison of the chelating properties of H_2_L^2−^ with similar hydroxyl bisphosphonate ligands shows that the strength of the Na–O(furanyl/hydroxyl) bond is directly related to the total charge of the ligand anion.

## 1. Introduction

Bisphosphonates (BPs) represent a very important class of compounds known for their medicinal and other properties [1, 2]. In contrast to the naturally occurring pyrophosphate P-O-P group, these compounds contain a characteristic P-C-P bridge, which is chemically and enzymatically nonhydrolyzable. BPs exhibit specific affinity towards bone, which makes them an excellent therapeutic for bone resorption diseases (especially osteoporosis, Paget's disease, tumour induced osteolysis, hypercalcemia originated from malignancy) by inhibition of farnesyl diphosphate synthase (FPPS) and for bone tumour caused by metastatic breast tumours [3–5]. The biological activity of bisphosphonates has been found to be dependent on the structure [6], lipophilicity [7], and bone binding affinity [8] of the compounds. For example, the structure of the side chain of bisphosphonates is important in determining the potency of individual bisphosphonates in biological models, and this includes a potential role for the side chain in modulating bone binding. The presence and position of an oxygen or nitrogen atom within the side chain of bisphosphonates are directly related to their relative potency and bone binding affinity. In general, the oxygen/nitrogen containing bisphosphonates exhibits high bone binding affinities, which might be expected to reduce efficacy. However, some of these molecules are very potent and the difference in potency has been attributed to the inhibition of FPPS. Therefore, the structural characterization of bisphosphonates is very important tool for the elucidation of the mechanisms of the bone binding and biological activity and for the design of new, more potent compounds. 

 Herein, we report the synthesis, solution characterization, and crystal structure of the sodium salt of tetrahydrofuranyl-2,2-bisphosphonate (Na_2_H_2_L). In the crystal the compound forms, the unusual for bisphosphonates, two-dimensional parallel sheets arrangement. A comparison of the chelating properties of H_2_L^2−^ with similar hydroxyl bisphosphonate ligands shows that the Na–O(furanyl/hydroxyl) bond length increases as the charge of the ligand decreases. Thus, the weak Na–O(furanyl) interaction is attributed to the −2 charge of the ligand. 

## 2. Materials and Methods

### 2.1. Materials

The chemicals obtained from commercial sources were reagents grade and used as received. Solvents were freshly distilled and water was doubly distilled. *γ*-Chlorobutyric acid, phosphorus acid, and D_2_O were purchased from Sigma-Aldrich. Sodium hydroxide and phosphorus trichloride were purchased from Merck. Ethyl alcohol and methyl alcohol were bought from BDH. The synthesis was performed in inert Argon atmosphere.

### 2.2. Synthesis and Crystallization of Tetrahydrofuranyl-2,2-Bisphosphonic Acid Disodium Salt (Na_2_H_2_L)

**Scheme 1:**
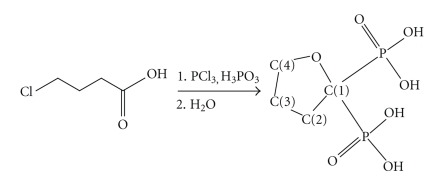
Synthetic methodology for synthesis of Na_2_H_2_L along with the numbering scheme of C-atoms for NMR peak assignments.

A mixture of *γ*-chlorobutyric acid (7.12 g, 58.1 mmol), phosphorus acid (3.38 g, 41.2 mmol), and phosphorus trichloride (10.9 g, 79.4 mmol) was heated slowly to reach the temperature 80°C over an hour under constant flow of argon. The two-phase mixture was refluxed for 4 hours at 80°C and the viscous yellow mass was stirred vigorously for 10 hours at 60°C. Precooled water (125 mL) was added slowly to the mixture under stirring as evolution of gaseous HCl was observed. The water (110 mL) was constantly removed by distillation from the orange reaction mass. Another 150 mL of water was added to the mixture and allowed to cool down slowly to room temperature (25°C). The pH of the reaction mixture was adjusted to 3 by careful addition of 4 M NaOH solution. Absolute ethyl alcohol (150 mL) was poured slowly to the resulting yellow solution in order to induce crystallisation. White solid was formed after 12 hours stirring at 25°C and collected by filtration. The yellow filtrate was again treated with ethyl alcohol (100 mL) to obtain another batch of white solid. Total three batches were collected and for the crude product the yield was calculated to be 9.75 g, (65% based on *γ*-chlorobutyric acid). Crystallisation was performed by layering concentrated aqueous solution (pH = 4.2) of Na_2_H_2_L with methyl alcohol. Samples for NMR measurements were prepared by dissolution of the crystals in D_2_O. The atoms have been numbered according to the numbering in Scheme 1. ^1^H NMR *δ*(D_2_O, ppm): 3.90 (t, ^2^ *J*_H,H_ = 6.7 Hz, two protons H(4)); 2.30 (m, ^2^ *J*_H,H_ = 6.7 Hz, ^3^ *J*_H,P_ = 16.0 Hz, two protons, H(2)); 1.99 (p, ^2^ *J*_H,H_ = 6.7 Hz, two protons H(3)). ^13^C NMR *δ*(D_2_O, ppm): 81.3 (t, ^1^ *J*_C,P_ = 144 Hz), C(1); 70.3, C(4); 30.2, C(3); 26.8 (t, ^2^ *J*_C,P_ = 2.26 Hz), C(2). ^31^P NMR *δ*(D_2_O, ppm): 21.9 (t, ^3^ *J*_H,P_ = 16.0 Hz). Anal. Calcd. for C_4_H_8_Na_2_O_7_P_2_ (f.w.: 276.02): C, 17.41; H, 2.92. Found: C, 17.38; H, 2.89. 

### 2.3. NMR Studies

The one-dimensional ^1^H, ^13^C, and ^31^P spectra were recorded in a 300 MHz Avance Bruker spectrophotometer at 300.13 MHz for ^1^H, 121.46 MHz for ^31^P, and 75.47 MHz for ^13^C with a 5 mm multinucleus probe at ambient temperature (25°C) in D_2_O solution. A 30°-pulse width and 1-second relaxation delay were applied for ^1^H, ^31^P, and ^13^C NMR spectra.

### 2.4. X-Ray Crystallography

Crystal data, details of data collection, and refinement of crystal structure of Na_2_H_2_L are provided in Table 1. The intensity data for the compound were collected at 100 K on *an XCalibur III 4-cycle diffractometer*, equipped with a CCD camera detector. The experimental data were collected using Mo K*α* radiation (*λ* = 0.7107 Å) at a crystal-to-detector distance 60 mm. The structure was solved by direct methods using the programs SHELXS-86 and refined anisotropically (nonhydrogen atoms) by full-matrix least squares on *F* ^2^ [9, 10]. The H atoms were calculated geometrically and refined with riding model with isotropic displacement parameters. The programs ORTEP-3 [11] and Diamond-3 were used for diagrams and WINGX [12] was used to prepare material for publication.

## 3. Results and Discussion

### 3.1. Synthesis

Compound Na_2_H_2_L has been synthesized by heating at 80°C the mixture of *γ*-chlorobutyric acid, phosphorus acid, and phosphorus trichloride (Scheme 1). This molecule has been previously synthesized by Kieczykowski et al. [13] following a similar procedure by heating the mixture at 65°C in methanesulfonic acid. However, the product isolated from that reaction had always been contaminated with the methanesulfonate salt and the yield of the reaction was less than 30%. In contrast, the reaction without the use of solvent gave pure product and yield more than 60%. Upon completion of the reaction, it is quenched with water. The product was crystallized by addition of ethanol in an aqueous solution of Na_2_H_2_L at pH 3.0. It is worth to notice that the isolated compound is the disodium salt of the bisphosphonate anion although it has been isolated at pH lower than that of the monosodium salt (pH 4.3) by Kieczykowski et al. This difference is attributed to the different methodology used for the crystallization of the product.

### 3.2. Solution Characterization

**Scheme 2:**
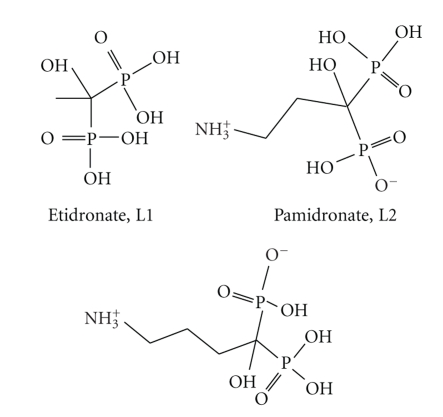
Molecular structures of etidronate, pamidronate, and alendronate and abbreviations used in the document.

The molecule was characterized in aqueous solution by ^1^H, ^13^C, and ^31^P NMR spectroscopy. The *J *coupling constants were used to confirm the assignments made by the NMR spectra. The ^31^P NMR spectrum gave one triplet due to the coupling (^3^ *J*_HP_ = 16.0 Hz) of phosphorus nuclei with the two H(2) protons at 21.9 ppm. This chemical shift is close to the chemical shifts of other hydroxyl bisphosphonates such as etidronate (L1, Scheme 2) (22.9 ppm) [14]. The ^13^C spectrum of Na_2_H_2_L showed a triplet assigned to C(1). The splitting of this peak is assigned to the strong coupling of C(1) with the two phosphorus atoms (^1^ *J*_CP_ = 144.0 Hz). The weak coupling of C(2) with the two phosphorus nuclei (^2^ *J*_C,P_ = 2.26 Hz) gives a triplet feature for this peak that can be easily distinguished from the peak assigned to C(3). The ^1^H NMR spectrum exhibits a triplet for H(4) protons (due to the coupling with the two H(3) protons), a quintuplet for H(3) (due to the coupling with the two H(2) and two H(4) protons), and a multiplet for H(2) (due to the coupling with the two H(3) protons and the two P atoms). 

### 3.3. X-Ray Crystallographic Structure

**Figure 1:**
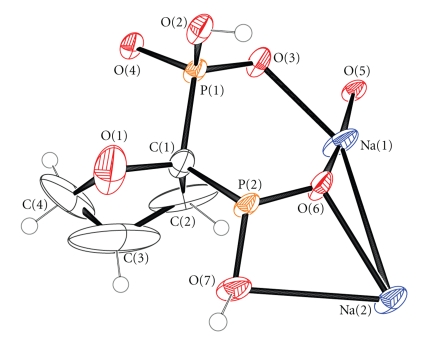
ORTEP representation of the main structural motif observed in Na_2_H_2_L. Ellipsoids are drawn at the 50% probability level.

**Figure 2:**
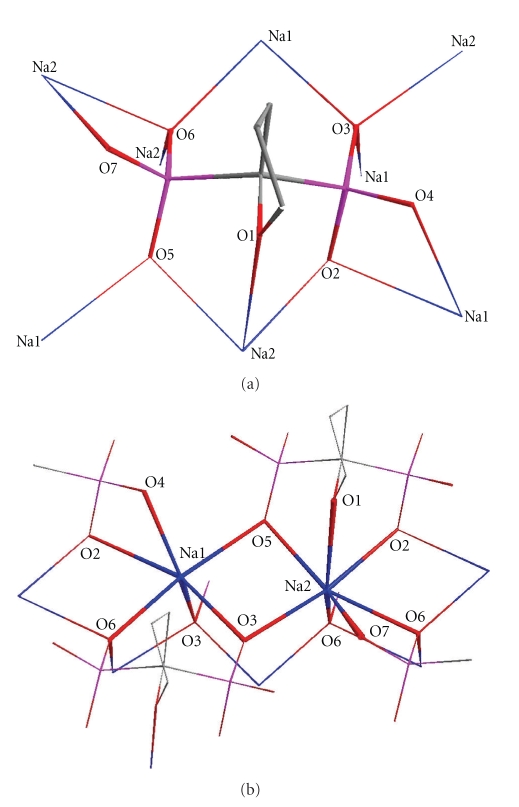
(a) Stick representation of H_2_L^2−^ showing the connectivity with sodium ions. (b) Stick representation of Na_2_H_2_L showing the coordination environment of sodium ions.

The molecular structure of Na_2_H_2_L is shown in Figures 1 and 2(a). Crystallographic data are provided in Table 1. Tables 3 and 4 contain the interatomic bond lengths and angles of Na_2_H_2_L. The bisphosphonate anion in the structure of Na_2_H_2_L possesses an overall −2 charge, thus two of the phosphonate oxygen atoms are deprotonated (P–O^−^), two have protons attached (P–OH), and two form double bond with phosphorous (P=O). Both phosphorous atoms exhibit distorted tetrahedral geometries. The P–O bond lengths are close to 1.50 Å for the P-O(deprotonated) and 1.57 Å for the P–OH(protonated). Although the P–OH bonds can be located very easily by inspection of the bond distances, the P–O^−^ and P=O cannot be distinguished because of the charge delocalization over the ^−^O–P=O groups. The bond angles around P(1), P(2) range from 104.0(4) to 116.4(3)°. The O–P–O angles involving the two unprotonated oxygen atoms are the largest ones in both phosphonate groups. The two phosphorus centers are fairly close (3.087(3) Å), due to their bisectional positions relative to C(1) on the furanyl ring (P(1)–C(1)–P(2) = 114.7(5)°). The P^…^P distance is a little larger than the distance (3.035(3) Å) observed in the crystal structure of nitrogen containing respective molecule, the pyrrolidine-2,2-diylbisphosphonic acid [15].

**Figure 3:**
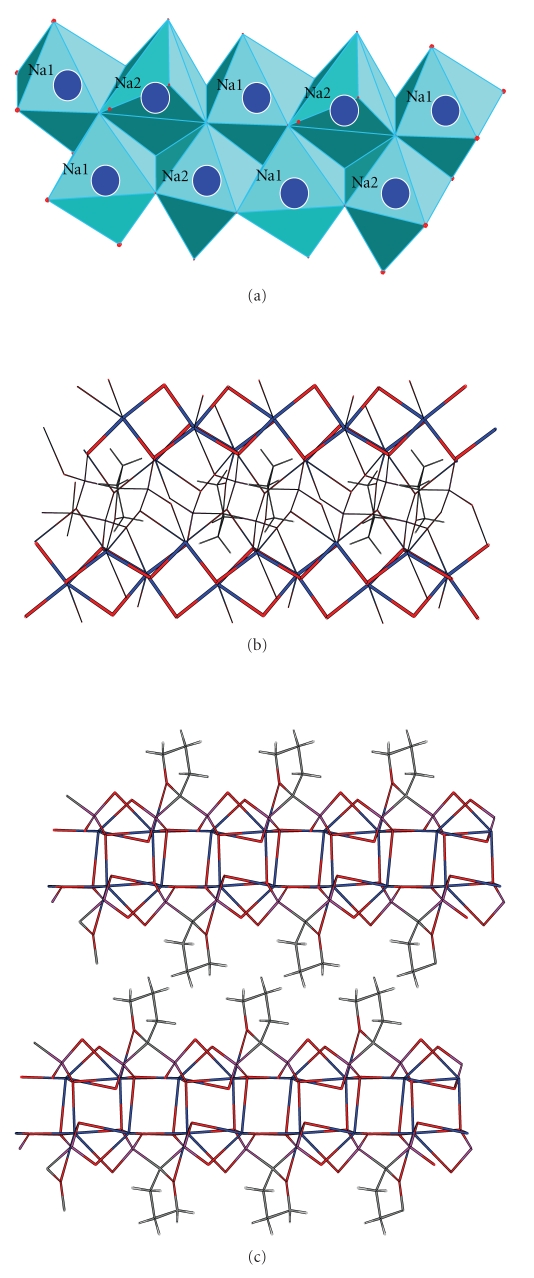
(a) Sodium-centered polyhedral connected to each other to form linear arrays along axis (1,1,0). (b) Connection of the linear sodium arrays with bisphosphonate anions to form two dimensional sheets. (c) Two layers stacked one parallel to the other along axis *c*.

The coordination sphere is different for each of the two sodium atoms (Figures 3(a) and 3(b)). The Na(1) exhibits a distorted octahedral environment defined by O(5), O(4), O(2), O(6), O(3), and O(3′). The Na(2) exhibits a capped octahedral geometry with the O(1), O(2), O(3), O(5), O(6), and O(6′) to define an octahedron and the O(7) added to the triangular face defined by O(1), O(3), and O(6). The Na–O bond distances range between 2.327(7) and 2.872(7) Å with the Na(2)–O(7) to exhibit the longest bond distance. The sodium-centered octahedrons and capped octahedrons form an edge-sharing linear array directed along the axis (1,1,0) of the crystal (Figure 3(a)). These arrays are connected to each other with the diphosphonate anions (Figure 3(b)) forming infinite 2D sheets. The two-dimensional networks are stacked one over the other along axis *c* (Figure 3(c)). The distance between the mean planes defined from the atoms of two successive sheets is 11.668(5) Å. This distance is actually the length of the *c* axis. The furanyl rings are located above and below the 2D structures insulating the sodium ions in each layer. It is possible that this unusual for diphosphonates two-dimensional structure is induced by the hydrophobic furanyl rings which prevent any electrostatic interlayer interactions. 

A recent study [23] has shown that bisphosphonates binding strength can be calculated by summarizing the interactions of the phosphonate groups, the side chain groups, the hydroxyl group, and the hydrophobic group of the ligand with the bone surface. Most importantly, it has been found that although the −OH group is necessary for the strong binding of phosphonates on the bone, the interaction of the −OH with the bone surface is weak. Selected bond lengths of Na^+^–O/Ca^2+^–O of similar tetrahydrofuranyl-2,2-bisphosphonate compounds extracted from their crystal structure are shown in Table 4. The comparison shows that the chelating properties of the ligands are in good agreement with their binding properties on bone surface. Thus, the bond distances between the donor oxygen atoms of bisphosphonate ligand and the cations might give an estimation of the bisphosphonates bone binding strength. The (Na^+^/Ca^2+^)–O(phosphoryl) bond distances are similar, within the range 2.261 to 2.873 Å, suggesting that the interaction of all phosphonate groups with the bone is of similar strength for all ligands. In all crystal structures of the calcium salts of the bisphoshonates, except the structure of etidronate, the metal ion is ligated only to the phosphonate oxygen atoms. The charge of the ligand in all calcium structures is −2. In contrast, the Na^+^ ions have been found to be ligated both to the phosphonate and the hydroxyl oxygen in the structures of the sodium salts of the respective ligands. However, the Na^+^–O bond length is much shorter in the structures of monoanion bisphosphonate structures (~2.5 Å) than in the respective dianions (~ 2.8 Å) including Na_2_H_2_L. Apparently, this bond length comparison indicates that the Na^+^–O(hydroxyl/furanyl) bond strength is directly dependent on the total charge of the bisphosphonate ligand; the bond length increases by decreasing the charge of the ligand. In addition, the weak interaction of hydroxyl group with the bone [23] also suggests that the total charge of the ligand that binds the bone surface is −2. 

The Na^+^–O bond distances found in the crystal structure of Na_2_H_2_L are close to the respective bond distance of Na_2_H_2_L2 (L2 = pamidronate, Scheme 2) showing that the contribution of these bonds for bone binding is similar [20]. However, the tetrahydrofuranyl side chain does not contain any group that will contribute to the bone binding, thus it is expected that H_2_L^2−^ will be a weaker bone binder than H_2_L1^2−^, H_2_L2^2−^, and H_2_L3^2−^ (Scheme 2).

## 4. Conclusions

The tetrahydrofuranyl-2,2-bisphosphonic acid has been prepared with an efficient method without the use of solvent, producing pure product in high yield over 60%. The crystal structure of the complex showed that the disodium salt of bisphosphonate crystallizes forming two-dimensional sheets stacked parallel one over the other.

The results of this study show that the crystallographic characterization of a widely used class of drug molecules, the bisphosphonate salts, provide important information on their biological activity (bone binding, FPPS inhibition), and can be used for the design of new more active molecules. The bisphosphonates binding strength with bone can be calculated by summarizing the interactions of the phosphonate groups, the side chain groups, the hydroxyl group, and the hydrophobic group of the ligand with the bone surface. The crystallographic data of the Na^+^/Ca^2+^ salts of bisphosphonate show that the interaction of the –OH group with the metal ions is weak which is in agreement with the results from a recent study [23] on the interaction of the bisphosphonates with bone surface. Furthermore a comparison of Na^+^/Ca^2+^ bisphosphonate structures shows that the strength of the Na–O(hydroxyl/furanyl) bond reduces with the decrease of the total bisphosphonate anion charge. 

## Figures and Tables

**Table 1:** Crystallographic and experimental data for compound Na_2_H_2_L.^a, b^

Empirical formula	C_4_H_6_Na_2_O_7_P_2_
Formula mass	274.01
Crystal system	Triclinic
Space group	*P-1*
*a*/Å	6.327 (5)
*b*/Å	6.967 (5)
*c*/Å	11.805 (5)
*α*/°	93.067 (5)
**β**/°	96.222 (5)
*γ*/°	113.594 (5)
*V*/Å^3^	471.4 (5)
*Z*	2
*D*_c_/g cm^−3^	1.930
Absorption coefficient/cm^−1^	0.564
*F* (000)	276
*θ* range for data collection/°	3.7788–62.3061
	−7 ≤ *h* ≤ 7
Ranges for *h*, *k*, *l *	−8 ≤ *h* ≤ 7
	−11 ≤ *h* ≤ 13
Reflections collected/unique	2384/1451
*R*_int _	0.0218
Data/restraints/parameters	3667/0/171
GOF on *F* ^2^	1.158
*a*, *b* in weighting scheme	0.1390/0.0668
Final *R*/*R*_w_ indices (*I* > 2*σ*(*I*))	0.0572/0.2162
Final *R*/*R*_w_ indices (all data)	0.0827/0.2288

^a^All structures determined at *T* = 100 K using Mo *K* *α* radiation (*λ* = 0.71073 Å).

^b^ *R* = Σ||*F*_o_|−|*F*_c_||/Σ|*F*_*o*_|, *w* *R* = [Σ*w*(|*F*_*o*_|^2^−|*F*_*c*_|^2^)/Σ*w*|*F*_*o*_|^2^]^1/2^, GOF = [Σ[*w*(*F*_*o*_^2^−*F*_*c*_^2^)^2^]/(*n*−*p*)]^1/2^, *w* = 1/[*σ* ^2^(*F*_*o*_^2^) + (*a* *P*)^2^ + *b* *P*], where *P* = (*F*_*o*_^2^ + 2*F*_*c*_^2^)/3.

**Table 2:** Selected bond lengths (Å) for Na_2_H_2_L.

C(1)–O(1)	1.48 (2)	P(1)–O(2)	1.571 (8)
C(1)–C(2)	1.50 (2)	P(1)–O(3)	1.499 (7)
C(2)–C(3)	1.59 (3)	P(1)–O(4)	1.499 (5)
C(3)–C(4)	1.50 (4)		
C(4)–O(1)	1.42 (2)	P(2)–O(5)	1.507 (7)
C(1)–P(1)	1.834 (9)	P(2)–O(6)	1.493 (7)
C(1)–P(2)	1.833 (7)	P(2)–O(7)	1.567 (7)

Na(1)–O(2)	2.625 (7)	Na(2)–O(1)	2.79 (2)
Na(1)–O(3)	2.335 (7)	Na(2)–O(2)	2.494 (8)
Na(1)–O(3′)	2.671 (8)	Na(2)–O(3)	2.371 (7)
Na(1)–O(4)	2.449 (8)	Na(2)–O(5)	2.405 (7)
Na(1)–O(5)	2.480 (7)	Na(2)–O(6)	2.423 (8)
Na(1)–O(6)	2.327 (7)	Na(2)–O(6′)	2.458 (6)

^a^Standard deviations are given in parentheses.

^b^Symmetry operations: *x*, *y*, *z*; −*x*, −*y*, −*z*.

**Table 3:** Selected bond angles (°) for Na_2_H_2_L.

O(2)–P(1)–O(3)	110.7 (3)	O(5)–P(2)–O(6)	115.1 (3)
O(2)–P(1)–O(4)	105.5 (3)	O(5)–P(2)–O(7)	110.1 (4)
O(3)–P(1)–O(4)	116.4 (4)	O(6)–P(2)–O(7)	109.3 (4)
C(1)–P(1)–O(2)	105.2 (4)	C(1)–P(2)–O(5)	108.6 (4)
C(1)–P(1)–O(3)	110.9 (4)	C(1)–P(2)–O(6)	109.2 (4)
C(1)–P(1)–O(4)	107.4 (4)	C(1)–P(2)–O(7)	104.0 (4)
O(1)–C(1)–P(1)	106.5 (6)	O(1)–C(1)–P(2)	105.2 (6)
C(2)–C(1)–P(1)	107.9 (8)	C(2)–C(1)–P(2)	110.7 (8)
O(1)–C(1)–C(2)	112.0 (9)	P(1)–C(1)–P(2)	114.6 (5)
C(1)–O(1)–C(4)	105 (1)	C(1)–C(2)–C(3)	100 (1)
O(1)–C(4)–C(3)	107 (2)	C(2)–C(3)–C(4)	103 (2)

^a^Standard deviations are given in parentheses.

^b^Symmetry operations: *x*, *y*, *z*; −*x*, −*y*, −*z*.

**Table 4:** Selected bond lengths for Na^+^ and Ca^2+^ bisphosphonate complexes. The L, L1, L2, L3 bisphosphonates are according to Scheme 2.

Compound	Charge of bisphosphonate anion	M–O_hydroxy/furanyl_/Å	Shortest M–O_phosporyl_/Å	Longest M–O_phosporyl_/Å	References
Na_2_H_2_L	(−2)	2.79 (1)	2.327 (7)	2.873 (8)	This work
CaH_2_L1	(−2)	2.608 (2)	2.352 (1)	2.608 (2)	[16]
NaH_3_L1	(−1)	2.470 (4)	2.230 (4)	2.447 (4)	[17]
NaH_3_L1	(−1)	2.463 (2)	2.293 (3)	2.444 (2)	[18]
CaH_2_L2	(−2)	^a^	2.288 (1)	2.387 (1)	[19]
Na_2_H_2_L2	(−2)	2.828 (3)	2.274 (3)	2.691 (3)	[20]
NaH_3_L2	(−1)	2.537 (2)	2.261 (2)	2.438 (2)	[21]
CaH_2_L3	(−2)	^a^	2.304 (1)	2.345 (1)	[22]

^a^ Non bonding distance above 3 *Ǻ*.
